# Feasibility of in vivo whole heart DTI and IVIM with a 15 minute acquisition protocol

**DOI:** 10.1186/1532-429X-16-S1-O15

**Published:** 2014-01-16

**Authors:** Martijn Froeling, Gustav J Strijkers, Aart J Nederveen, Steven A Chamuleau, Peter R Luijten

**Affiliations:** 1 Department of Radiology, University Medical Center, Utrecht, Netherlands; 2 Department of Biomedical Engineering, Biomedical NMR, Eindhoven University of Technology, Eindhoven, Netherlands; 3 Department of Radiology, Academic Medical Center, Amsterdam, Netherlands; 4 Department of Cardiology, Division Heart & Lung, University Medical Center, Utrecht, Netherlands

## Background

In recent years in vivo cardiac DTI using stimulated echo's (STE) has matured into a reproducible technique. However the STE approach requires two heartbeats and intrinsically has a 50% lower SNR compared to spin-echo (SE). Although the STE method allows for short TE (23 ms) it also suffers from T1 signal decay and typically 8 signal averages (16 heartbeats) are needed for a single slice acquisition. In this study we aimed to develop a SE-based cardiac diffusion MRI protocol that allows for whole heart DTI as well as intra-voxel coherent motion (IVIM) for perfusion assessment.

## Methods

**Figure 1:**
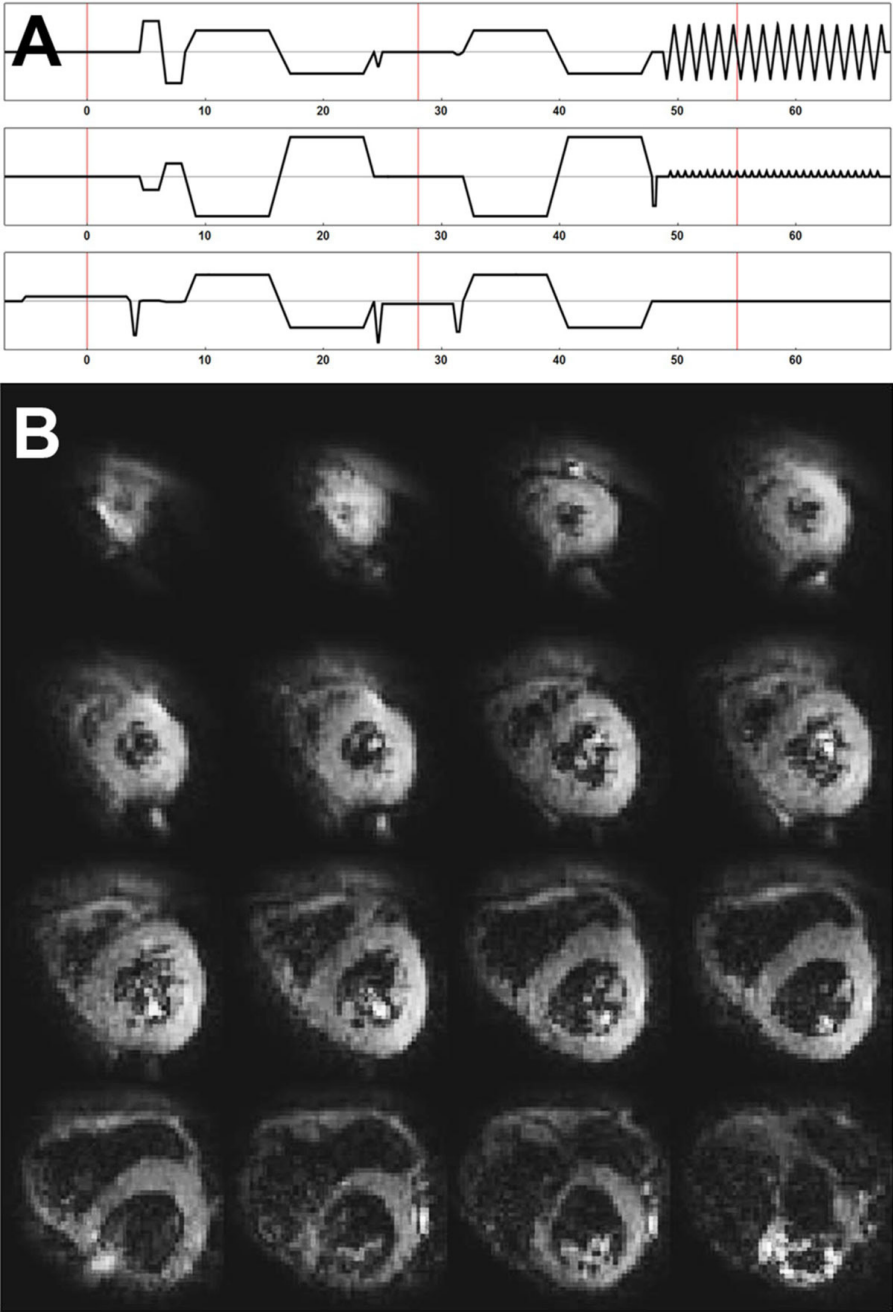
**A) Diffusion-weighted SE sequence with bipolar diffusion encoding and flow compensation gradients directly after the 90 degree slice selection**. B) The acquired single shot diffusion weighted data for b = 300 s/mm^2^, with a voxel size of 6 × 2.5 × 2.5 mm^3 ^and TE = 55 ms

Images were acquired with cardiac triggering (200 ms) and free breathing on a 3T scanner (Philips, Achieva) using a 16-channel coil (Torso XL). DWI was performed using a SE sequence with bipolar diffusion weighting gradients and additional flow compensation (Figure 1A). A reduced FOV was obtained using outer volume suppression. The diffusion weighting gradients were applied in 3 orthogonal directions with for b-values of 30, 60, 90, 120 s/mm^2 ^and in 12 directions for a b-value of 300 s/mm^2^. Additionally 4 non-weighted images were acquired resulting in 28 volumes. Every volumes was acquired twice resulting in a total acquisition time of 15 min for a heart rate of 60 bpm. Further parameters were; FOV:280 × 150 mm^2^, voxel size: 6 × 2.5 × 2.5 mm^3^, slices: 16, BW-EPI: 42 Hz TR: 8 heartbeats, TE: 55 ms. First data was registered to correct for heart- and breathing motion using a 2D non-rigid method followed by Rician noise suppression. Finally data was fitted to: S(b, g) = S0((1-fr) exp(-b g **D **g^T^)+ fr exp(-b g **D **g^T ^D*)) using a constrained non-linear least squares method. Fiber tractography was performed the vIST/e toolbox with a step size of 0.2 voxel. Stopping criteria were 0.1 < FA < 0.6 and an angle change of 20° per step.

## Results

**Figure 2:**
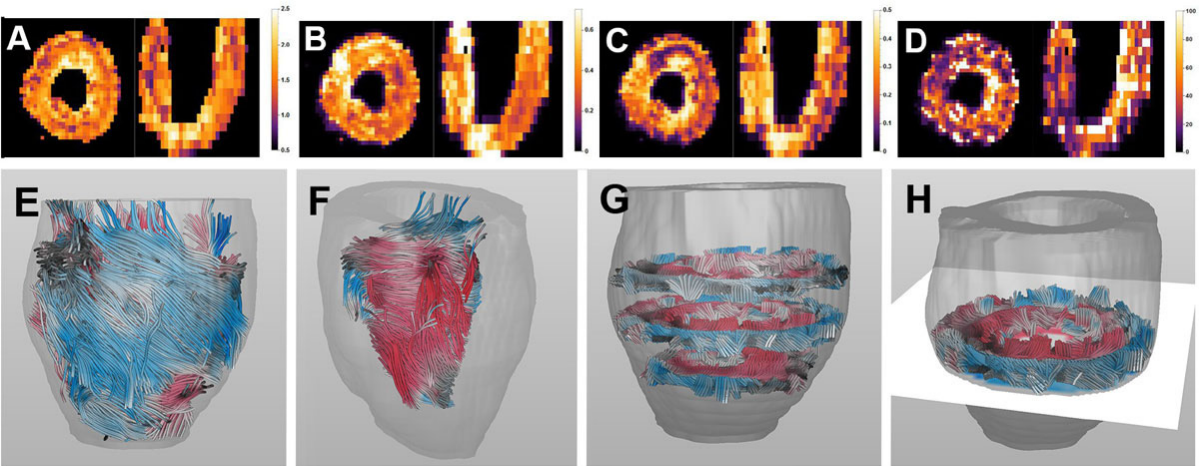
A-D) Parameter maps based on the IVIM fit (A: MD in 10-3 mm^2^/s, B: FA, C: fraction, D: D* in in 10-3 mm^2^/s). E-F) whole heart fiber tractography based on the IVIM tensor fit color coded for helix angle. (E: whole heart, F: Inside of the myocardial wall with papillary muscle, G-H: local fiber orientation for different cross sections)

The corrected DWI images for b = 300 s/mm^2 ^are shown in Figure 1B. Figure 2A to 2D show parameter maps for MD, FA, f and D* resulting from the combined IVIM and tensor fit. The average values for the whole heart were 1.67 ± 0.49*10^-3 ^mm^2^/s, 0.46 ± 0.20, 0.27 ± 0.16, 52.68 ± 52.61*10^-3 ^mm^2^/s respectively. The cardiac helical fiber organization could be reproduced by fiber tractography as shown in Figure 2E to 2G where the fiber tracts are color coded for the helix angle.

## Conclusions

In this study we have shown that it is feasible to acquire whole heart DTI and IVIM data within a 15 min protocol in free breathing. Using this approach we were able to quantify the diffusion and perfusion and visualize the fiber architecture.

## Funding

Not applicable.

